# Trichoscopy of twisted hairs in a patient with unilateral ecchymoses: A case of modern-day scurvy

**DOI:** 10.1016/j.jdcr.2026.05.064

**Published:** 2026-06-05

**Authors:** Camilla A. Cascardo, Severine Cao

**Affiliations:** Michigan Medicine Department of Dermatology, Ann Arbor, Michigan

**Keywords:** cork-screw hairs, ecchymosis, nutritional deficiency, scurvy, vitamin C deficiency

## Introduction

Vitamin C deficiency (scurvy), once considered rare, has reemerged in recent years, particularly among individuals with highly selective diets and neurodevelopmental disorders.^1^^,^^2^ Early recognition is essential, as the condition is readily treatable but may mimic infectious, vascular, or rheumatologic disease. This case highlights trichoscopic findings of “twisted” hair shafts, an atypical hair shaft abnormality, underlining the utility of trichoscopy in diagnosing scurvy. Additionally, our case highlights the role of mechanical factors in unilateral distribution of findings, emphasizing the variability in scurvy presentation.

## Case report

A man in his 20s with a history of autism and attention-deficit/hyperactivity disorder presented to the Emergency Department with a 1-month history of progressive right lower extremity swelling, stiffness, pain, and ecchymosis. The ecchymoses initially involved the right calf and later extended to the posterior thigh. He denied fevers, chills, night sweats, trauma, or prior abnormal bleeding but reported frequently resting his leg over the armrest of his couch. A dietary history revealed a highly restrictive diet consisting almost exclusively of meats and grains. Despite outpatient evaluation and treatment, his symptoms continued to worsen, prompting hospital admission for further workup and subsequent dermatology consultation.

**Fig 1:**
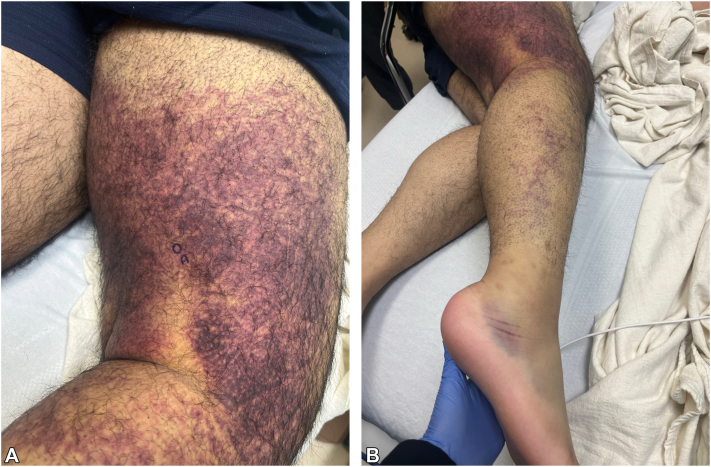
**A** and **B,** Ecchymoses and associated induration on the right lower extremity.

**Fig 2:**
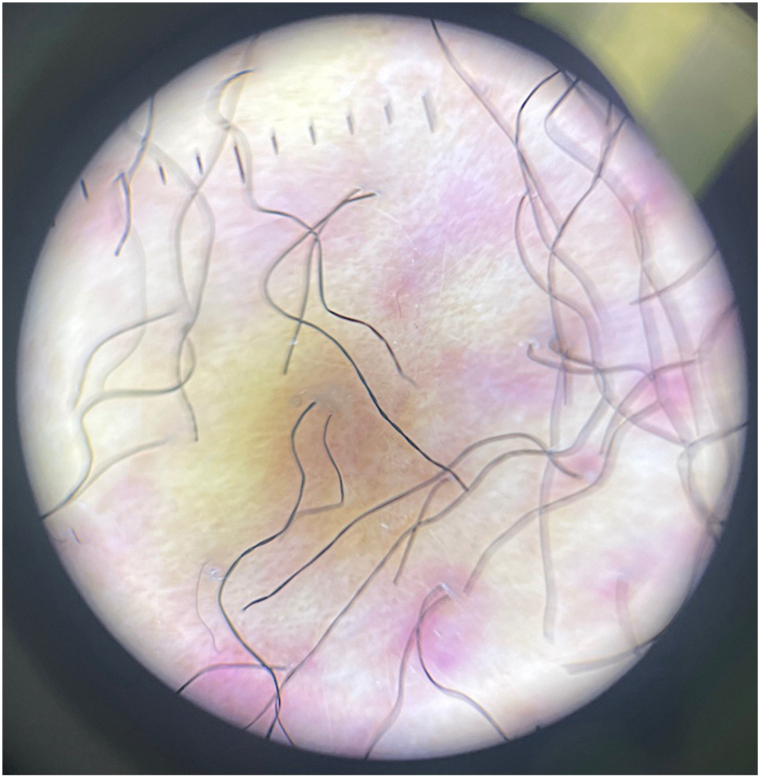
Perifollicular hemorrhage and twisted hairs highlighted with trichoscopy.

Physical examination demonstrated right lower extremity edema with overlying ecchymoses and associated induration (Fig 1, *A* and *B*). Trichoscopy revealed numerous twisted hair shafts on the affected extremity (Fig 2), including loosely coiled and irregularly bent hairs rather than classic tightly wound corkscrew hairs. Scattered perifollicular hemorrhage was present on both lower extremities and, to a lesser extent, the upper extremities with keratosis pilaris-like appearance. Gingival telangiectasias were also observed. No lymphadenopathy was appreciated.

Initial outpatient evaluation included duplex venous ultrasound and computed tomography angiography, which demonstrated no evidence of deep venous thrombosis or active bleeding. Laboratory evaluation demonstrated normocytic anemia with reticulocytosis, elevated lactate dehydrogenase, lymphopenia, elevated D-dimer, and indirect hyperbilirubinemia, consistent with hemorrhage. Coagulation studies, antinuclear antibody testing, and serum free light chain analysis were unremarkable. Magnetic resonance imaging revealed extensive perimysial and intramuscular edema involving the posterior calf and distal thigh musculature without necrosis or abscess, suggestive of a myositis-like process.

**Fig 3:**
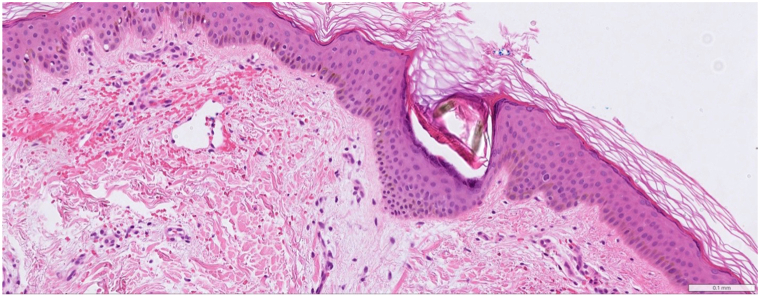
Magnification 20×; Superficial dermal hemorrhage with a coiled hair shaft.

A punch biopsy demonstrated superficial, largely non-inflammatory dermal hemorrhage with sparse perivascular lymphocytes and a coiled hair shaft (Fig 3). There was no evidence of leukocytoclastic vasculitis, vascular proliferation, or vascular malformation.

Given the constellation of perifollicular purpura, ecchymoses, gingival findings, hair shaft abnormalities, restrictive diet, and supportive histopathology, scurvy was suspected. The patient was empirically started on oral vitamin C supplementation (250 mg daily) prior to laboratory confirmation. Serum vitamin C level subsequently returned at <0.1 mg/dL, confirming severe deficiency. He was discharged with outpatient nutritional counseling, and several weeks later, experienced complete resolution of bruising and symptoms.

## Discussion

Vitamin C (ascorbic acid) is essential for collagen synthesis, serving as a cofactor for prolyl and lysyl hydroxylases that stabilize the collagen triple helix.^3^^,^^4^ Deficiency results in impaired collagen cross-linking, capillary fragility, and defective connective tissue, manifesting as hemorrhage, impaired wound healing, and systemic symptoms including anemia.^3^^,^^4^

Cutaneous findings are often the earliest diagnostic clues and include perifollicular hemorrhage, ecchymoses, follicular hyperkeratosis, and characteristic hair abnormalities such as corkscrew hairs.^1^ Perifollicular hemorrhage and ecchymoses arise from structural weakness of dermal capillaries, while follicular hyperkeratosis and corkscrew hairs reflect abnormal follicular support.^3^^,^^5^ Gingival bleeding similarly reflects collagen-dependent vascular fragility. In this patient, the combination dermatologic and mucosal findings provided key diagnostic direction in the setting of an otherwise broad differential.

Hair abnormalities represent a particularly useful but underrecognized diagnostic feature of scurvy. While corkscrew hairs are classically described, this patient demonstrated twisted, lightly curled and irregularly bent hair shafts on trichoscopy rather than the classic tightly coiled corkscrew hairs. Twisted hairs and other hair shaft abnormalities, including “swan-neck” deformities, have been described in scurvy, although dermoscopic documentation remains limited.^6^ Our case highlights the trichoscopy finding of an underreported hair shaft abnormality and emphasizes the diagnostic utility of trichoscopy in suspected nutritional deficiencies, particularly when presentations are atypical.

Twisted hair shafts may represent a spectrum of hair shaft deformities seen in vitamin C deficiency, influenced by mechanical forces, hair length, or deficiency stage. The appearance is reminiscent of pili torti, which is characterized by flattened shafts rotated along their longitudinal axis with regularly spaced twists, and is associated with numerous congenital and acquired conditions.^7^ Whether the twisted hairs seen in scurvy represent true pili torti or a phenotypic mimic unique to the pathophysiology of vitamin C deficiency warrants further study.

Musculoskeletal manifestations of scurvy include myalgias, hemarthroses, and intramuscular hemorrhage.^8^^,^^9^ Imaging findings of diffuse myofascial edema, as seen here, may mimic inflammatory myositis or infection contributing to diagnostic uncertainty and delay in treatment.^8^^,^^9^ These findings can prompt extensive vascular and rheumatologic evaluation, particularly when the presentation is unilateral or progressive. The unilateral distribution in this case may have been influenced by habitual limb positioning, suggesting a role for mechanical factors in localizing hemorrhagic manifestations in the setting of vascular fragility.

Treatment consists of vitamin C supplementation, typically 100 to 500 mg daily, with rapid clinical improvement.^3^^,^^8^ Early recognition prevents unnecessary diagnostic testing and prolonged morbidity. This case highlights scurvy as an important and potentially underrecognized cause of unilateral ecchymoses and musculoskeletal symptoms in patients with restrictive diets.^2^^,^^10^ Atypical hair shaft abnormalities on trichoscopy and mechanical factors influencing lesion distribution may provide additional diagnostic clues. Recognition of these features can facilitate timely diagnosis and effective treatment.

### Declaration of generative AI and AI-assisted technologies in the writing process

None.

## Conflicts of interest

None disclosed.
